# Molecular imaging biomarkers for immune checkpoint inhibitor therapy

**DOI:** 10.7150/thno.38339

**Published:** 2020-01-01

**Authors:** Pim P van de Donk, Laura Kist de Ruijter, Marjolijn N Lub-de Hooge, Adrienne H Brouwers, Anthonie J van der Wekken, Sjoukje F Oosting, Rudolf SN Fehrmann, Derk Jan A de Groot, Elisabeth GE de Vries

**Affiliations:** 1 Department of Medical Oncology, University Medical Center Groningen, University of Groningen, Groningen, Netherlands.; 2 Department of Clinical Pharmacy and Pharmacology, University Medical Center Groningen, University of Groningen, Groningen, Netherlands.; 3 Department of Nuclear Medicine and Molecular Imaging, University Medical Center Groningen, University of Groningen, Groningen, Netherlands.; 4 Department of Pulmonary Medicine, University Medical Center Groningen, University of Groningen, Groningen, the Netherlands.

**Keywords:** molecular imaging, biomarkers, positron emitting tomography, immune checkpoint inhibitor, immunotherapy.

## Abstract

Immune checkpoint inhibitors (ICIs) have substantially changed the field of oncology over the past few years. ICIs offer an alternative treatment strategy by exploiting the patients' immune system, resulting in a T cell mediated anti-tumor response. These therapies are effective in multiple different tumor types. Unfortunately, a substantial group of patients do not respond to ICIs. Molecular imaging, using single-photon emission computed tomography (SPECT) and positron emission tomography (PET), can provide non-invasive whole-body visualization of tumor and immune cell characteristics and might support patient selection or response evaluations for ICI therapies. In this review, recent studies with ^18^F-fluorodeoxyglucose-PET imaging, imaging of immune checkpoints and imaging of immune cells will be discussed. These studies are until now mainly exploratory, but the first results suggest that molecular imaging biomarkers could have a role in the evaluation of ICI therapy.

## Introduction

Since the approval of ipilimumab by the Food and Drug Administration (FDA) and European Medicines Agency (EMA) in 2011, immune checkpoint inhibitors (ICIs) have substantially changed the field of oncology. Monoclonal antibody (mAb) based therapies targeting cytotoxic T-lymphocyte antigen 4 (CTLA-4), programmed cell death 1 (PD-1) or programmed cell death ligand 1 (PD-L1) have improved patient survival across various tumor types 1-8. ICI therapies target the ability of cancer cells to evade the patient's immune system through disruption of inhibitory ligand-receptor interactions. This allows effector T cells to recognize and eradicate tumor cells. Currently, seven ICIs have been approved for clinical use by the FDA and EMA. These are the anti-CTLA-4 antibody ipilimumab, the anti-PD1 antibodies nivolumab, pembrolizumab and cemiplimab, and the anti-PD-L1 antibodies atezolizumab, avelumab and durvalumab. These antibodies are currently used to treat multiple tumor types including: melanoma, hepatocellular carcinoma, small-cell lung cancer, non-small-cell lung carcinoma (NSCLC), renal cell carcinoma, urothelial carcinoma, Hodgkin lymphoma, head and neck squamous cell carcinoma (HNSCC), Merkel cell carcinoma, gastric cancer, primary mediastinal large B-cell lymphoma and cervical cancer. Moreover, the FDA approved pembrolizumab and nivolumab as tumor agnostic therapy for patients with microsatellite instability-high (MSI-H) or deficient DNA mismatch repair (dMMR) tumors. This list of indications has been steadily growing as research progresses.

Despite this progress, a substantial group of patients does not respond to ICI therapy. A cross-sectional analysis of US patients with cancer eligible for ICI therapy for registered indications estimated a response rate of 12.46% in 2018 9. This unfortunately means that even for registered indications, only a minority of patients gain long term survival benefit from ICI therapy. Even though ICIs are generally well tolerated, they can cause immune-related adverse events (irAE). Higher response rates have been reported when ICIs are combined, but this coincides with an increase and different kinetics of irAEs 10, 11. Therefore, there is a need for reliable predictive biomarkers to either select patients at baseline for ICI therapy or to evaluate treatment efficacy early during therapy. Identifying which patients will benefit from these therapies would greatly improve patient care. Several biomarkers have been studied for ICI therapy. Currently, PD-L1 expression measured using immunohistochemistry (IHC) and MSI-H and dMMR status measurement by IHC and polymerase-chain-reaction based assays are the only approved biomarkers for ICI therapy. However, the assay for PD-L1 expression is hampered by multiple variables involved in tumor tissue analyses, such as: sampling errors, spatial heterogeneity or temporal heterogeneity of tumor characteristics 12-14.

Molecular imaging with single-photon emission computed tomography (SPECT) and positron emission tomography (PET), using specific radiopharmaceuticals, might potentially circumvent some of these issues. These techniques allow for non-invasive whole-body visualization of tumor and immune cell characteristics. Uptake of molecular imaging tracers can be quantified, and these measurements permit the technique to generate biomarkers. Since tumor characteristics, such as PD-L1 expression or tumor infiltrating lymphocyte numbers, can change over time, serial scans might provide information about dynamics of these aspects 13, 15.

Extensive research is being conducted to study the feasibility of molecular imaging biomarkers for ICI therapy. Regarding biomarkers, we adhere in this review to the terminology and definitions as posed by the FDA-NIH Biomarker Working Group and *O'Connor et al.* 16, 17. An imaging biomarker is defined as “a spatially delineated biomarker derived from measurements made on an image” 16. Quantification of tracer uptake, expressed as standardized uptake values (SUV), and anatomical imaging measurements can both serve as a biomarker. In this review, recent advances in the development of molecular imaging biomarkers for ICI therapies with the focus on molecular imaging approaches in clinical development will be discussed.

## Search strategy

PubMed was searched for relevant publications. Articles were selected when they were: published in peer reviewed journals, written in English and were available in full text. ClinicalTrials.gov was queried for relevant clinical trials investigating molecular imaging approaches for ICI therapies. The 2019 conference abstracts of the American Society of Clinical Oncology (ASCO) and the American Association of Cancer Research (AACR) were searched for relevant new developments. These databases were searched up to May 2019. The following key words were used in the literature search: molecular, imaging, immunotherapy, checkpoint, inhibitor, immune, positron emitting tomography OR PET, single-photon emission computed tomography OR SPECT, programmed cell death protein 1 OR PD-1, programmed death-ligand 1 OR PD-L1, cytotoxic T-lymphocyte-associated protein 4 OR CTLA-4, lymphocyte, tumor, cancer, CD8, CD4, CD3. Specific search terms for isotopes included (zirconium OR Zr-89 OR ^89^Zr), (copper OR cu-64 OR ^64^Cu), (fluorine OR F-18 OR ^18^F), (gallium OR Ga-68 OR ^68^Ga), (iodine OR I-124 OR ^124^I), (yttrium OR y-86 OR ^86^y), (carbon OR c-11 OR ^11^C), (technetium OR Tc-99m OR ^99m^Tc).

## Response evaluation of ICIs

Currently, the Response Evaluation Criteria in Solid Tumors (RECIST) version 1.1, for which the criteria were validated on a data warehouse, are used to determine response to chemotherapeutic drugs. These criteria are based on anatomical imaging measures 18. However, ICI therapy can result in temporary pseudo-progression. Therefore, the RECIST working group developed a guideline for data collection with a modified version of RECIST v1.1 for immune based therapeutics termed iRECIST 19. The major change being that in case of progression, while the patient's condition is not deteriorating, the progression has to be confirmed a few weeks later to prove progressive disease during ICI. The data, collected using these guidelines, will be used to define the iRECIST criteria, once sufficient studies have been analyzed.

## ^18^F-FDG-PET imaging

The^ 18^F-fluorodeoxyglucose (^18^F-FDG)-PET scan, which visualizes increased cell metabolism with ^18^F-radiolabeled glucose-like FDG in tumors and sites of inflammation, is the most frequently used type of PET scan in oncology. It is mainly used for staging of patients with cancer. ^18^F-FDG-PET is included in RECIST v1.1, but only to confirm progressive disease when indicated. Right now, not enough data is available to validate ^18^F-FDG-PET response criteria by the RECIST committee on a data warehouse. Several groups are exploring the use of ^18^F-FDG-PET scans for predicting response to ICI therapy (Table 1). Twelve studies, totaling 468 patients, evaluated the ^18^F-FDG-PET in patients with melanoma, NSCLC and Hodgkin's lymphoma 20-31. In these studies, metabolic responses are defined as a decrease in SUV in the tumor. The results do suggest an association between metabolic responses and clinical outcomes. However, multiple criteria were applied for ^18^F-FDG-PET response evaluations, such as the EORTC criteria, PERCIST, PERCIMT, PECRIT and iPERCIST 24, 26, 29, 32, 33. Moreover, ^18^F-FDG-PET imaging was performed at different time points after starting ICI therapy, making comparisons difficult. For ^18^F-FDG-PET, to obtain a potential role for tumor response measurement during ICI therapy, standardized and harmonized procedures and analyses should be applied. Generating comparable data would allow for further analyses to define the role of ^18^F-FDG-PET in ICI therapies. Such a thorough analysis will be critical as changes in glucose metabolism can be caused by various cell types in the tumor microenvironment, including tumor cells, immune cells or stromal cells.

## Imaging of immune checkpoints

### PD-1:PD-L1 axis

The PD-1 receptor is expressed by immune cells, mainly by activated T cells 34. It functions as an immune checkpoint. Binding to its ligand, PD-L1, results in inhibition of T cell activation. PD-L1 is expressed by various other immune cells, including dendritic cells, T cells and non-lymphoid parenchymal tissue cells 35. PD-L1 expression is upregulated upon cytokine release induced by T cell activation and an inflammatory environment. The PD-1:PD-L1 immune checkpoint is involved in maintaining immunological tolerance, and thus reducing auto-immunity and healthy tissue damage. Tumor cells expressing PD-L1, escape destruction by the immune system. Patients with high PD-L1 expressing tumors, determined by IHC, tend to show better overall survival (OS) when treated with ICI compared to those with PD-L1 negative lesions. However, patients without PD-L1 tumor expression can respond to therapy 36-38. PD-L1 expression by tumor cells is dynamic and can be upregulated by ICI therapy and by inflammatory cytokines, such as interferon gamma 34, 39. Furthermore, tumor biopsies do not take into account the spatial heterogeneity of PD-L1 target expression, which can highly differ within and across metastases within one patient 15, 40-47.

## PD-1:PD-L1 imaging with full antibodies

Full mAbs are large proteins, generally around 150 kDa. Their weight and structure causes slow tissue distribution after intravenous administration and prevents filtration by the kidneys, resulting in an elimination half-life around 18-21 days 48. For imaging purposes, optimal PET imaging with full mAb tracers requires several days to allow the antibody to accumulate at the target location. Acquisition of the optimal PET images generally takes place 4-7 days after tracer injection.

**Figure 1:**
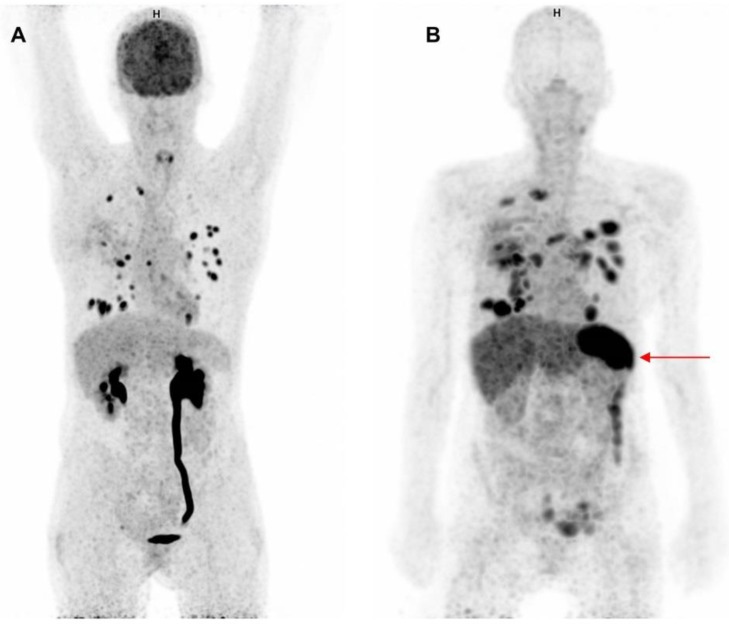
**Comparison of ^18^F-FDG-PET and ^89^Zr-atezolizumab-PET imaging.** Maximum intensity projections of ^18^F-FDG-PET, 1 h after tracer injection (A) and ^89^Zr-atezolizumab-PET, 7 days after tracer injection (B) of a 53 year old woman diagnosed with NSCLC. Both imaging modalities show uptake in multiple intra-pulmonary metastases. The ^18^F-FDG-PET scan shows physiological high uptake in the brain and excretion via the kidneys. At the moment of this scan the patient had a post-renal obstruction due to a kidney stone. The ^89^Zr-atezolizumab-PET scan shows high uptake in the spleen (red arrow). The ^18^F-FDG-PET scan was performed 46 days prior to the ^89^Zr-atezolizumab-PET scan. Both scans were scaled equally (0-8).

Two clinical studies have been published in which therapeutic anti-PD1 or anti-PD-L1 mAbs have been radiolabeled for PET imaging of the PD-1:PD-L1 checkpoint. This approach can provide information about their tumor penetration and target engagement. One trial was performed with 10 mg of the PD-L1 antibody atezolizumab radiolabeled with zirconium-89 (^89^Zr) and PET imaging on days 4 and 7 in patients with NSCLC, cancer of the urinary tract, and triple negative breast cancer prior to atezolizumab treatment (figure 1) 49. ^89^Zr-atezolizumab tumor uptake was positively associated with a response rate to atezolizumab treatment. Moreover, the geometric mean uptake of ^89^Zr-atezolizumab above or below median correlated to progression free survival (PFS) and OS. For individual lesions, higher ^89^Zr-atezolizumab tracer uptake is associated with tumor size reduction over time. In contrast, PD-L1 expression in tumor biopsies, obtained 7 days after tracer injection and immediately after the last PET scan, did not correlate with response to therapy. Tracer uptake was also seen in lymphoid tissues such as normal lymph nodes, tonsils and the spleen. Since PD-1:PD-L1 inhibitors induce a systemic immune response with effects throughout the body, tracer uptake measured with PET in normal tissues might also serve as a proxy for evaluation of activation of the immune system. Uptake of the tracer was also seen in sites of inflammation, including a sinusitis and a knee bursitis. Another study reported high ^89^Zr-atezolizumab uptake in a mouse bearing a tumor graft model of a patient with metastatic clear cell renal cell carcinoma. This patient later appeared to have a durable response to nivolumab treatment 50.

In another study a PD-1 antibody, namely 2 mg nivolumab was radiolabeled with ^89^Zr and PET imaging was performed 7 days after tracer injection in 13 patients with NSCLC prior to nivolumab treatment 51. Uptake of ^89^Zr-nivolumab was analyzed for 21 tumor lesions: uptake was higher in the 7 lesions that showed a reduction in size of ≥30% at 12 weeks after start with nivolumab treatment. ^89^Zr-nivolumab uptake correlated with PD-1 expression by tumor-infiltrating immune cells assessed by IHC, in fresh baseline tumor biopsies or archival tissue. Also, spleen tracer uptake was seen with a SUV of 5.8, which is much lower than the spleen uptake seen with ^89^Zr-atezolizumab 49. In the ^89^Zr-nivolumab study, five out of 13 patients responded to the therapy: the study provides a rationale for evaluation of ^89^Zr-nivolumab in a larger cohort to evaluate its potential as a tool for response prediction.

All currently approved PD-1:PD-L1 targeting antibodies have been radiolabeled with ^89^Zr 49, 51-54. This radionuclide with radioactive half-life (78.4 hours) suits the long half-life of the antibodies and their tendency to slowly distribute in the body. Radiolabeled tracers of registered anti-PD-L1 mAbs, such as ^89^Zr-avelumab (NCT03514719), ^89^Zr-durvalumab (NCT03610061, NCT03829007) and ^89^Zr-atezolizumab (NCT02453984, NCT03850028) are currently investigated in clinical trials. Most of these studies perform PET imaging prior to ICI therapy to evaluate tracer uptake in the tumor lesions as a predictive biomarker. Another multicenter trial in patients with thoracic malignancies recently started with ^89^Zr-labeled REGN3504, a non-registered anti-PD-L1 antibody (NCT03746704). ^89^Zr-atezolizumab PET imaging is also performed serially during atezolizumab therapy to evaluate tumor saturation by atezolizumab (NCT02453984).

Two trials with the PD-1 antibody ^89^Zr-pembrolizumab are ongoing (NCT02760225, NCT03065764), both performing PET imaging before starting ICI therapy.

## PD-1:PD-L1 imaging with small molecules and antibody fragments

Smaller molecules have been engineered for imaging of PD-1 and PD-L1 55-57. These agents are in general not designed to induce a therapeutic effect, but to enable early imaging. An exception is the PET tracer ^89^Zr-KN035 55. KN035, a 79.6 kDa domain antibody targeting PD-L1, is evaluated in several clinical trials for its therapeutic potential. The ^89^Zr-labeled domain antibody is studied to evaluate the biodistribution and lesion uptake of the tracer in patients with PD-L1 positive advanced solid tumors (NCT03638804).

In the above-mentioned clinical trial with ^89^Zr-nivolumab 51, they also studied a radiolabeled anti-PD-L1 adnectin (^18^F-BMS-986192) with a molecular weight around 10 kDa 58, 59. Fluorine-18 (^18^F) with a radioactive half-life of 110 minutes resembles the elimination half-life of small molecules like adnectins. PET acquisition was performed 60 minutes after ^18^F-BMS-986192 injection. ^18^F-BMS-986192 uptake was higher in the three patients with PD-L1 expression of ≥50% determined by IHC. However, higher tracer uptake did not correlate with response to nivolumab treatment in this small cohort.

A single domain antibody (sdAb) NM-01 with a molecular weight of ~15 kDa, which binds human PD-L1 was labeled with technetium-99m (^99m^Tc) for SPECT imaging in patients with NSCLC prior to anti-PD-L1 antibody treatment 59. In an early analysis of the first 16 patients, the tracer showed heterogeneous tumor uptake between patients, between metastases and within tumors. No data is reported on correlation with uptake of the tracer and outcome of therapy. Besides uptake in the spleen and bone marrow, the authors do not report on specific uptake of this PD-L1 tracer in other normal lymphoid tissues. The trial is ongoing and aims to recruit 50 patients (NCT02978196).

Compared to the full antibodies agents ^89^Zr-atezolizumab 49 and ^89^Zr-nivolumab 51, clearance of ^99m^Tc-NM-01 is faster, resulting in mean tumor:bloodpool ratios of 1.79 (1.24-2.3) at 1 hour after injection and 2.33 (1.24-3.53) at 2 hours after injection. ^89^Zr-atezoluzimab PET showed the highest tumor:bloodpool ratio around 5.0 on day 7. Overall, ^89^Zr-atezolizumab uptake in tumors had geometric mean SUV_max_ 10.4. Imaging of ^89^Zr-nivolumab at 7 days after tracer injection yielded a mean SUV_peak_ of 6.4 in responding lesions versus 3.9 in non-responding lesions; uptake of ^18^F-BMS986192 1 hour after injection showed similar uptake values. However, no information about contrast to background tissue and bloodpool are provided, making it difficult to interpret the imaging results and uptake values of the tracers.

The peptide WL12 (14-amino acid circular peptide), engineered to bind PD-L1 with high affinity, was radiolabeled with copper-64 (^64^Cu) to allow for PET imaging 60. Although only tested in mouse models, WL12 shows the potential for imaging with small molecules. The binding interface of WL12 to PD-L1 overlaps with that of its natural receptor PD-1 and all registered PD-L1 antibodies. Therefore, the authors suggest that it may be used to evaluate unoccupied PD-L1, providing information on drug-target engagement for prediction of ICI therapy efficacy. Performance of the tracer in mouse models did indeed demonstrate reduction of ^64^Cu-WL12 uptake in tumors after atezolizumab treatment. This outcome was consistent in tumors with different expression levels of PD-L1, and dose and time dependent changes could be quantified. Analyses of tissue penetration of a mAb may be of interest. In a study with ^89^Zr-trastuzumab, a mAb against human epithelial growth factor 2 (HER2), 29% of the patients with tumors expressing HER2 expression by IHC showed no uptake of ^89^Zr-trastuzumab in their tumors 61. Apparently, penetration of a drug into tumor tissue does not solely rely on target presence. An ongoing ^89^Zr-atezolizumab study (NCT02453984) addresses this issue by repeating imaging during atezolizumab treatment. Head-to-head comparisons of the imaging performance of mAbs versus smaller PD-L1 binding moieties are still lacking.

Reported results on quantification of tracer uptake differ between all reported tracers targeting the PD-1:PD-L1 axis. Overall, ^89^Zr-atezolizumab showed the highest SUV in tumor tissue compared to the other clinically evaluated tracers ^89^Zr-nivolumab, ^18^F-BMS986192 and ^99m^Tc-NM-01.

## Imaging of CTLA-4

Ipilimumab, which blocks checkpoint molecule CTLA-4, was the first ICI in the clinic in 2011. CTLA-4 is expressed by activated T cells and regulatory T cells (T_regs_); expression levels are upregulated after binding of the T cell receptor with antigen on antigen presenting cells 34. Ligands for CTLA-4 are B7-1 and B7-2 on antigen presenting cells, for which CTLA-4 competes with the stimulatory molecule CD28 on T cells. Engagement of the ligands with CTLA-4 causes an inhibitory signal for T cell activation, due to its competition with CD28 and prevention of ligand binding to CD28.

Currently one clinical trial (NCT03313323) is investigating CTLA-4 imaging with ^89^Zr-ipilimumab as a predictive biomarker for ipilimumab therapy. ^89^Zr-ipilimumab uptake is quantified upfront and early during treatment with ipilimumab, with the idea that patients who do not benefit from ipilimumab treatment may have lower levels of the drug in tumor tissues.

In immunocompetent mice, an anti-mouse ^64^Cu-DOTA-anti-CTLA-4 mAb was used to visualize CTLA-4 levels in tissues 62. The mouse tumor cell line expressed little CTLA-4, but when growth was induced, these tumors showed high tracer uptake on the ^64^Cu-DOTA-anti-CTLA-4 PET scan, which correlated with higher influx of tumor infiltrating lymphocytes. In contrast, little uptake of the tracer was seen in tumors in immunodeficient mice.

In another study CTLA-4 PET imaging was performed in a humanized mouse model 63. ^64^Cu-NOTA-ipilimumab and ^64^Cu-NOTA-ipilimumab-F(ab')_2_, a full antibody and an antibody fragment, localized CTLA-4+ engrafted human peripheral blood lymphocytes. These preclinical results suggest that it is feasible to perform PET imaging with anti-CTLA-4 tracers to visualize CTLA-4.

## Upcoming targets in checkpoint inhibition

As developments proceed, more checkpoint molecules are becoming subject of research, including lymphocyte activation gene 3 (LAG-3), T-cell immunoglobulin and mucin-domain containing-3 (TIM3), T cell immunoreceptor with Ig and ITIM domains (TIGIT) and V-domain Ig suppressor of T cell activation (VISTA) 34, 64, 65. Currently, anti-LAG-3 antibodies are most extensively studied, with 50 ongoing trials registered at ClinicalTrials.gov. LAG-3, a checkpoint molecule that is upregulated on activated T cells, is considered to be a marker for T cell exhaustion. LAG-3 binds to major histocompatibility complex-II (MHC-II), thus preventing binding of the T cell receptor to MHC-II 66. LAG-3 PET imaging has been performed in mice. ^89^Zr-REGN3767 visualized LAG-3 expressing intratumoral T cells after co-implantation with human lymphoma cells and human peripheral blood mononuclear cells 67. ^89^Zr-LAG-3 PET is currently studied in patients with NSCLC and HNSCC prior to anti-LAG-3 therapy (NCT03780725).

Not only inhibitory immune checkpoints are being investigated. Costimulatory checkpoint molecules, such as OX40 (CD134), inducible T cell costimulator (ICOS, CD278), glucocorticoid-induced TNFR-related protein (GITR), 4-1BB (CD137), CD40 and CD27 have been identified as potential therapeutic targets. For OX40 68 and CD40 69, radiotracers targeting these cell surface proteins have been developed.

## Imaging of T cells

Immunotherapy can potentiate the T cell mediated immune response against tumor cells. Therefore, molecular imaging of T cells has great potential for the evaluation of new and current therapies. Visualizing T cells might allow us to evaluate the anti-tumor immune response early and could potentially support adaptations of the treatment regimen.

As the immune checkpoint molecules CTLA-4, PD-1 and PD-L1 are present on T cells, imaging with tracers targeting the checkpoint interactions generates information on presence, activation and migration of T cells. Moreover, imaging of T cells can also be performed with tracers against T cell specific targets, independent of checkpoint molecules. Cluster of differentiation 8 positive (CD8+) T cells are generally considered to be the main effector cells involved in the immune response against tumor cells. Several tracers have been developed to visualize CD8+ T cells. Radiolabeled antibody constructs can detect changes in tumor infiltrating lymphocytes following ICI therapy in mice 70-73. Two CD8 PET tracers are studied clinically: a phase I trial using a zirconium labeled mini-body construct (⁸⁹Zr-Df-IAB22M2C) has been completed (NCT03107663) and is now evaluated in a phase II trial (NCT03802123). The preliminary results of the phase I trial showed no tracer related adverse effects, cytokine release syndrome or blood test abnormalities. The anti-CD8 tracer accumulated in CD8-rich tissues, such as the spleen, bone marrow and lymph nodes. Tracer uptake by the tumor was variable and seen in 10 of the 15 patients 74. Another zirconium labeled anti-CD8 imaging agent ZED88082A is currently studied in patients that receive ICIs (NCT04029181).

Another approach is the use of radiolabeled interleukin-2 (IL2) for lymphocyte imaging. The high affinity IL2 receptor, which consists of three subdomains (CD25, CD122 and CD132) is highly expressed by several lymphocyte subtypes, such as T_regs_ and activated T cells. The low affinity IL2 receptor, which consists of two subdomains (CD122 and CD132) is found on naive T cells, memory T cells and natural killer cells 75. A first proof of concept study was performed in five patients with metastatic melanoma using ^99m^Tc labeled interleukin-2 (^99m^Tc-IL2) 76. Patients were scanned at baseline and 12 weeks after starting treatment. In two out of three patients that underwent an on-treatment ^99m^Tc-IL2 PET scan, a lower total SUV_max_ was seen in the tumor lesions after treatment, while one patient had an increase in total SUV_max_. A ^18^F labeled IL2 tracer is being evaluated in melanoma patients (NCT02922283). Patients are scanned at baseline and during ICI therapy to determine whether changes in tumor tracer uptake correlate with response to ICI therapy.

^18^F labeled 9-β-d-arabinofuranosylguanine ([^18^F]F-AraG) is also investigated as a PET tracer for T cell imaging in cancer patients. [^18^F]F-AraG is a positron-emitting guanosine analog that selectively accumulates in T cells. [^18^F]F-AraG can be phosphorylated by cytoplasmic deoxycytidine kinase and deoxyguanosine kinase and is subsequently trapped intracellularly 77, 78. This tracer is evaluated in stage I-IIIa NSCLC patients receiving neoadjuvant pembrolizumab with or without radiotherapy (NCT03311672). A single [^18^F]F-AraG PET scan is performed after 2 cycles pembrolizumab. Results will be compared to CD3+ T cell numbers present in the resection specimens.

It is a challenge for T cell imaging to determine the optimal imaging time point to capture the T cell influx, as the time it takes to detect major T cell accumulation in the tumor microenvironment following ICI therapy is not yet precisely known. Lymphocyte activation, proliferation and functional differentiation can occur in a matter of days following viral infection 79. Whether this also applies for ICI therapy in cancer is unknown. T cell influx early during treatment has been investigated by obtaining early on-treatment biopsy samples and by studying resection specimen in neoadjuvant ICI therapy studies after treatment (Table 2). This data suggests that an increase in T cell numbers already occurs within a few weeks after the start of treatment 80-86. T cell imaging in this timeframe could provide a non-invasive way to visualize T cell dynamics after only one or two doses of ICI therapy.

Another challenge for T cell imaging is the complexity of the human immune system, which consists, among others, of many lymphocyte subgroups which play different roles in the immune response. Some of these subgroups are pro-inflammatory or effector cells. Other subgroups, such as T_regs_ are anti-inflammatory and have been associated with worse OS and resistance to therapy 87, 88. Unfortunately, the abovementioned PET-tracers do not specifically target one uniform subgroup of T cells. The targets of these tracers can be expressed by both effector T cells as well as anti-inflammatory T cell subsets. At the same time, these tracers do not visualize all effector- or anti-inflammatory T cell subsets. For example, a tracer directed against CD8 only shows the CD8-mediated immune response. However, multiple different effector T cells have been implicated to play a role in the anti-tumor immune response. Some natural killer cells and γδ T cells do not express CD8 and therefore this part of the anti-tumor immune response might be overlooked during CD8 imaging 89, 90.

## Imaging of other immune components of the tumor microenvironment

Understandably, most effort is focused on imaging of effector T cells to monitor the efficacy of immunotherapies. However, imaging of other immune cells might also offer valuable information regarding the dynamic tumor microenvironment. Besides effector immune cells, there are several immune cell types present in the tumor microenvironment, such as T_regs_, tumor associated macrophages (TAMs) and myeloid-derived suppressor cells (MDSCs) 91. These cells play a key role in preserving the immunosuppressive state of the tumor microenvironment. To better understand the underlying mechanisms of resistance to ICI therapies, these cells types are of interest. Furthermore, a small subset of patients experiences early tumor progression after starting ICI therapy 92, 93. It is hypothesized that mechanisms associated with resistance to ICI therapy could be involved in hyperprogressive disease 93. This might be due to a shift towards a more immunosuppressive tumor microenvironment by proliferation or modulation of immunosuppressive cells, such as T_regs_, M2 TAMs or MDSCs 94-96. Visualizing these immunosuppressive cells might be useful to distinguish pseudo-progression from hyperprogressive disease.

At this moment, no T_reg_ specific molecular imaging tracers are available. However, there are tracers that target both T_regs_ as well as other immune cell subgroups. T_regs_ are generally characterized as CD4+ CD25+ FoxP3+ T cells. Radiolabeled anti-CD4 antibody constructs have been developed and are currently investigated for future application in the clinic 97, 98. Furthermore, T_regs_ constitutively express high levels of the IL2 receptor 75. Therefore, IL2 imaging might have a potential role in T_reg_ imaging. Future studies will have to determine whether these tracers are effective at imaging T_regs_ and whether this holds any diagnostic value for visualizing resistance to therapy or the evaluation of tumor progression. Multiple PET and SPECT tracers have been developed for imaging of macrophages 99, 100. These tracers are already being studied in diseases such as auto-immune diseases, atherosclerosis and cancer. However, imaging of macrophages has not yet been investigated for ICI therapies. MDSC imaging has been performed using ^99m^Tc‐anti‐CD11b SPECT imaging in a murine colon tumor model. Tracer uptake was seen in the tumor, spleen and bone marrow 101.

Not only immune cells can be visualized using molecular imaging. Cytokines also play an important role in the dynamics of the tumor microenvironment. Transforming growth factor beta (TGF-β) causes exclusion of T cells from the tumor microenvironment. This is associated with poor prognosis in cancer, and resistance to PD-L1 and PD-1 inhibitors. 102, 103. Currently, several trials are conducted with TGF-β inhibitors, some in combination with ICI, as preclinical findings have shown an increase in effector T cells in the tumor 103. PET imaging of TGF-β with the ^89^Zr-labeled antibody fresolimumab in patients with recurrent glioblastoma showed high tracer uptake in the tumor. This tumor type is known for high expression of TGF-β and its receptors 104. ^89^Zr-fresolimumab or other tracers visualizing TGF-β may enlighten resistance mechanisms to ICI therapy.

## Discussion

The developments in ICI therapies have resulted in remarkable tumor responses and improvements in patient survival. An effective immune response induced by ICI therapy is the result of an interplay between the antibody targeting checkpoint molecules, the tumor cells, and the patient's immune system. Molecular imaging has the potential to provide measurable imaging biomarkers that may predict effects of these therapies. Furthermore, radiolabeling of therapeutic mAbs informs researchers about the pharmacokinetic properties of these drugs.

Translating preclinical findings from animal tumor models to clinical results is challenging. A realistic representation of a human immunological response requires not only a human(-ized) tumor model with a human target, i.e. with expression of human PD-L1, but also human variants of all the co-players attributing to an effective cancer immune response 105. Moreover, most preclinical mouse studies are done in mice aged 6-8 weeks, whereas patients usually develop cancer at an older age. This could be relevant as the immune system and lymphoid system are subject to change as people get older 106.

The clinical studies discussed in this review are exploratory studies and provide only a glimpse of the tracer's potential value. The major challenge lying ahead is implementing these imaging approaches in patient care. This requires harmonization of procedures and proper validation in larger cohorts. Data sharing and collaboration will truly benefit this process and reduce costs 107. However, it is a challenge worth facing as the approaches discussed in this review show great potential to provide insight in the ICI mediated anti-tumor immune response.

## Figures and Tables

**Table 1:** Studies with ^18^FDG-PET for tumor response evaluation of checkpoint inhibitor therapy

Treatment	Tumor type	Number of patients	Response criteria	Imaging time points	Summary of findings	Reference
Ipilimumab	Melanoma	22	EORTC	^18^F-FDG-PET scan at baseline, after 2 cycles and after 4 cycles ipilimumab	^18^F-FDG-PET/CT after 2 treatment cycles is predictive of final treatment outcome in patients with progressive and stable disease	(20)
Pembrolizumab or nivolumab	Melanoma	27	Qualitative visual analysis	^18^F-FDG-PET scan on treatment (median after 15.2 months)	Negative ^18^F-FDG-PET scans may hold negative predictive value for disease progression	(21)
Ipilimumab	Melanoma	31	Fractal and multifractal analysis	^18^F-FDG-PET scan at baseline, and after 2 and 4 cycles ipilimumab	In 20 out of 24 cases (83.3 %), results of the fractal and multifractal analysis were consistent with treatment outcome data	(22)
Ipilimumab, nivolumab or BMS-936559 (anti-PD-L1 antibody)	Melanoma	20	RECIST 1.1 and PERCIST	^18^F-FDG-PET/CT at 4 weeks and at 4 months	^18^F-FDG-PET/CT scans predict response with 100% sensitivity, 93% specificity and 95% accuracy	(23)
Nivolumab	NSCLC	24	RECIST 1.1 versus PERCIST	^18^F-FDG-PET scan at baseline and at 1 month	Metabolic response (especially total lesion glycolysis) at 1 month is associated with response to nivolumab and survival	(24)
Checkpoint inhibitor	NSCLC	27	Semi-quantitative analysis (SUV_max_ and SUV_mean_)	^18^F-FDG-PET scan at baseline	SUV_max_ ≤17.1 (sensitivity 88.9%) or a SUV_mean_ ≤8.3 (sensitivity 100%) identifies fast progression after 8 weeks therapy	(25)
Ipilimumab	Melanoma	41	PERCIMT	Baseline and after 4 cycles ipilimumab	Four new ^18^F-FDG-avid lesions after 4 cycles ipilimumab is an indication of nonresponse	(26)
Ipilimumab	Melanoma	41	PERCIMT versus EORTC	Baseline and after 2 cycles ipilimumab	PERCIMT more sensitive predictor of clinical response to ipilimumab than EORTC criteria	(27)
Nivolumab or pembrolizumab as monotherapy or plus ipilimumab	Melanoma	104	RECIST and EORTC	Baseline and after 1 year	After 1 year of therapy, 68% patients with a partial response on CT scan had a complete metabolic response on ^18^F-FDG PET scan	(28)
Nivolumab	NSCLC	28	iPERCIST versus iRECIST	Baseline, after 2 months, and when warranted after 3 months	Comparison of iPERCIST to iRECIST showed reclassification of 39% of patients with relevant additional prognostic information	(29)
Nivolumab or pembrolizumab	Hodgkin lymphoma	43	Visual evaluation according to Deauville score and Lugano criteria	Baseline, after 8 weeks and after 17 weeks	No differences compared to the LYRIC criteria. Metabolic activity could be related to immune response as early as 8 weeks after start of treatment	(30)
Ipilimumab	Melanoma	60	PERCIST	Baseline and after completing ipilimumab treatment	Tumor response according to PERCIST associated with OS. This association improved when using modified response criteria (imPERCIST)	(31)

**Abbreviations**: EORTC: European Organization for Research and Treatment of Cancer; iPERCIST: immune PET Response Criteria in Solid Tumors; iRECIST: immune Response Evaluation Criteria in Solid Tumors; OS: overall survival; PERCIMT: PET Response Evaluation Criteria for Immunotherapy; PERCIST: PET Response Criteria in Solid Tumors; RECIST: Response Evaluation Criteria in Solid Tumors; SUV: standardized uptake value.

**Table 2:** Changes in tumor infiltrating lymphocytes following ICI therapy in serial tumor tissues measured immunohistochemically

Setting	Treatment	Tumor type	Number of patients	Sampling time points	Summary of findings	Reference
Neoadjuvant	2 cycles nivolumab 3 mg/kg body weight	NSCLC	21	Baseline and tumor resection after 4 weeks	Major pathological response in 45% of resected tumors	(80)
Neoadjuvant	2 cycles ipilimumab + nivolumab	Melanoma	10	Baseline and tumor resection after 6 weeks	Pathological response in 70% of biopsies	(81)
Neoadjuvant	1 cycle pembrolizumab	Melanoma	27	Baseline and tumor resection after 3 weeks	Complete or major pathologic response in 30% of patients. Increase in CD8 positive T cell numbers compared to pre-treatment biopsy	(82)
On-treatment biopsy	CTLA-4 blockade and/or PD-1 blockade	Melanoma	53	Baseline and early on treatment (after 2-3 doses)	Increase in CD8+ T cells	(83)
On-treatment biopsy	Pembrolizumab	Melanoma	46	- Baseline - 20-60 days - 80-120 days - 120 days	Increase in CD8 T cell density (cells/mm^2^) in responders. Stable CD8 numbers in non-responders	(84)
On-treatment biopsy	Pembrolizumab	Melanoma	53	Baseline and on treatment (median 74 days)	Increase in T cell frequency. Increase in CD8+ effector memory T cells in responders. No changes in T_reg_ frequencies	(85)
On-treatment biopsy	Anti-PD1 therapy	Melanoma	13	Baseline and early on treatment (14 days)	Significant expansion of CD8+ cells early during treatment. Higher CD8 T cell numbers were seen in responders	(86)

**Abbreviations**: CD8: cluster of differentiation 8; CTLA-4: cytotoxic T lymphocyte antigen 4; NSCLC: non-small-cell lung carcinoma; PD-1, programmed cell death 1; T_reg_: regulatory T cell.

## Abbreviations

[^18^F]F-AraG: ^18^F labeled 9-β-d-arabinofuranosylguanine
^18^F: Fluorine-18
^18^F-FDG: ^18^F-fluorodeoxyglucose
^64^Cu: copper-64
^89^Zr: zirconium-89
^99m^Tc: technetium-99m
CD: Cluster of differentiation
CTLA-4: cytotoxic T-lymphocyte antigen 4
dMMR: deficient DNA mismatch repair
EMA: European Medicines Agency
FDA: Food and Drug Administration
HER2: human epithelial growth factor 2
HNSCC: head and neck squamous cell carcinoma
ICI: immune checkpoint inhibitor
IHC: immunohistochemistry
IL2: interleukin-2
irAE: immune-related adverse event
LAG-3: lymphocyte activation gene 3
mAb: monoclonal antibody
MDSC: myeloid-derived suppressor cell
MHC-II: major histocompatibility complex-II
MSI-H: microsatellite instability-high
NSCLC: non-small-cell lung carcinoma
OS: Overall survival
PD-1: programmed cell death 1
PD-L1: programmed cell death ligand 1
PD-L2: programmed cell death ligand 2
PET: positron emission tomography
PFS: progression free survival
RECIST: Response evaluation criteria in solid tumors
SPECT: single-photon emission computed tomography
SUV: standardized uptake value
TAM: tumor associated macrophage
TGF-β: transforming growth factor beta
TIGIT: T-cell immunoreceptor with Ig and ITIM domains
TIM3: T-cell immunoglobulin and mucin-domain containing-3
T_regs_: regulatory T cells
VISTA: V-domain Ig suppressor of T cell activation
